# Review of "Brachytherapy Physics, Second Edition" Edited by Bruce R. Thomadsen, Mark J. Rivard and Wayne M. Butler

**DOI:** 10.1186/1475-925X-5-4

**Published:** 2006-01-18

**Authors:** Mohammad-Reza Movahed

**Affiliations:** 1 Department of Medicine, Division of Cardiology, University of California, Irvine Medical Center, Orange, California, USA

This book is intended for radiation and medical physicists, biomechanical engineers, radiation oncologists and clinicians who are involved in the assessment and treatment of patients requiring close-distance radiation, which is defined as "brachytherapy". This book is a very comprehensive description of brachytherapy with emphasis on physics. It is an excellent book for physicists and biomedical engineers on understanding the detailed principles of brachytherapy and equipment, and for radiation oncologists for a detailed description of radiation sources and dosing of cancer patients. It emphasizes the two most commonly used applications for oncological patients with prostate and gynecological malignancies.

It is less suitable for the oncologist who is looking for detailed clinical trials or patient selection and follow-up. It is even less suitable for the cardiologist who uses brachytherapy in patients with stent restenosis. The intravascular brachytherapy chapter describes the details for the treatment of instent restenosis but does not mention any clinical trial or indications for this technique in cardiac patients [1]. Furthermore, the cardiac application of this therapy is neglected and the radiofrequency ablation chapter does not even mention the use of this technique for the treatment of arrhythmias [2]. Therefore, this book has limited usefulness for the cardiologist. The Vascular Brachytherapy book written by Waksman would certainly be more suitable for cardiovascular physicians [3].

Chapters were written by leading scientists with excellent qualifications in the relevant subjects. The book contains 54 chapters divided into six major sections: Fundamentals, Dosimetry, Interstitial Fundamentals, Interstitial Applications, Intercavitary Applications for Gynecological Cancers and Unconventional Delivery Systems. The book contains many excellent diagrams and illustrations accompanied by a CD-ROM version of the book. The important strengths and weaknesses are summarized below:

The Fundamentals includes a comprehensive review of brachytherapy physics and radiobiology. It is a well-written chapter especially for medical physicists or radiation oncologists. For the clinician, the details could be somewhat overwhelming.

The dosimetry and interstitial fundamental sections contain excellent comprehensive explanations of dosing and instrumentation of brachytherapy techniques with diagrams and illustrations.

The other chapters contain details on clinical application of brachytherapy in mostly cancer patients. Depending on the cancers described in this book, the chapters markedly differ. For example, the chapters describing brachytherapy principles and treatment of prostate and gynecological malignancies are very comprehensive and detailed. On the other hand, the chapters explaining this technique in other oncological patients with brain, eyes, head and neck cancers are relatively smaller and less comprehensive. In general, this book is written from a physicist's point-of-view which is very comprehensive for the principle and technical aspects of brachytherapy. However, the clinical indications and algorithms for the use of this technique and the discussion about the clinical and outcome data are less comprehensive and even lacking in some of the smaller chapters.

The Textbook of Radiation Oncology certainly is more clinically oriented covering multiple treatment plans and biology of the tumors and utilization of specialized techniques [4]. Radiation Therapy Physics [5] is an alternative book for radiation oncologists and biomedical engineers with broader coverage of radiation treatment beyond brachytherapy including imaging in therapy planning, calibration protocols and computer applications.
